# Beneficial effect of xylose consumption on postprandial hyperglycemia in Korean: a randomized double-blind, crossover design

**DOI:** 10.1186/s13063-016-1261-0

**Published:** 2016-03-15

**Authors:** Yun Ju Jun, Jinhee Lee, Sehee Hwang, Jung Hyun Kwak, Hyeon Yeong Ahn, Youn Kyung Bak, Jihoon Koh, Jong Ho Lee

**Affiliations:** Department of Food and Nutrition, National Research Laboratory for Clinical Nutrigenetics/Nutrigenomics, Yonsei University, Sudamun-Gu, Seoul Republic of Korea; Department of Food Science and Biotechnology, CHA University, Pocheon-si, Gyeonggi-do Republic of Korea; College of Pharmacy, Chung-Ang University, Dongjak-gu, Seoul Republic of Korea; Department of Preventive Medicine, Gachon University College of Medicine, Yeonsu-gu, Incheon Republic of Korea; Department of Food and Nutrition, Research Institute of Science for Aging, Yonsei University, Seodaemun-gu, Seoul Republic of Korea; Food Ingredients R&D Center, CJ Cheiljedang Co., Suwon-si, Gyeonggi-do Republic of Korea

**Keywords:** Xylose, Prediabetes, Postprandial hyperglycemia, Glucose

## Abstract

**Background:**

Previous studies have reported that xylose selectively inhibited the activity of sucrase. Xylose supplementation may have a beneficial effect on the postprandial glycemic response. However, no studies have investigated patients with IFG or the effectivity of a dose of D-xylose less than 10 % (w/w).

**Methods:**

The present study determined the effect of xylose consumption on postprandial hyperglycemia in normal (n = 25) and hyperglycemic subjects (n = 50). Subjects in this double-blind crossover design study were randomly assigned to consume a sucrose drink (Control, sucrose 50 g + deionized water 100 g) or a sucrose drink additionally containing 5 g (Test 1, sucrose:xylose = 10:1), 3.33 g (Test 2, sucrose:xylose = 15:1), or 2.5 g (Test 3, sucrose:xylose = 20:1) of D-xylose separated by a one-week interval.

**Results:**

Normal subjects in all test groups exhibited a significant decrease in serum glucose levels 15 min and 30 min after consuming the xylose-containing drinks compared to the control group. Significantly lower serum levels of insulin were observed at 15 min and 30 min after consuming the xylose-containing drinks compared to the control group. The test 1 group also exhibited a significantly lower insulin area under the curve than the control group. Hyperglycemic subjects (n = 50) in all test groups exhibited a significant decrease in serum glucose levels at 30 min compared to the control group. However, the test 1 group exhibited a significant increase in serum glucose levels at 120 min compared to the control group. Glucose-related markers did not significantly differ in each group.

**Conclusion:**

Xylose supplementation may exert a beneficial effect on postprandial glycemic responses in subjects with normal glucose levels and prediabetes.

**Trial registration:**

ClinicalTrials.gov identifier: NCT02654301. Registered 12 January 2016.

## Background

Data from the 2010 Korea National Health and Nutrition Examination Survey (KNHANES) reported that the dietary total saccharide intake (61.4 g/day) increased by 23 % compared to the 2008 KNHANES report [1]. The prevalence of impaired fasting glucose (IFG) in Koreans aged greater than 30 years has also increased consistently in the past three years [2–4]. Elevated postprandial blood glucose (PPBG) has been associated with an increased risk of metabolic disorders, type 2 diabetes, and cardiovascular disease (CVD) [5]. Therefore, strategies to reduce PPBG are very important.

Sugar is the most widely and commonly used sweetener worldwide, and various sweeteners, such as sugar alcohols, aspartame, and acesulfame potassium, were developed to substitute for sugar, the consumption of which is a potential reason for the rise in obesity and diabetes rates [6]. However, sugar alcohols cause cramping, bloating, and diarrhea in excessive amounts [7], and aspartame may be harmful to patients with phenylketonuria because of the generation of phenylalanine after consumption [8]. Therefore, these alternative sweeteners are limited as sugar substitutes.

Sucrase hydrolyzes sugar (sucrose) to glucose and fructose, which are rapidly absorbed into the blood via facilitated diffusion and active transport. Glucose absorption into the blood promotes insulin secretion, which promotes blood glucose migration into cells, generates energy, or promotes storage in the liver and muscle as glycogen [9]. However, the increase in blood glucose by sugar intake may burden the pancreas of subjects with IFG, and the consequent pancreatic damage reduces insulin secretion [10].

Xylose is a naturally occurring monosaccharide that is primarily found in wood, and D-xylose selectively inhibits sucrase activity. Therefore, D-xylose could prevent the rise in blood glucose and insulin, which would also decrease the burden on the pancreas [11].

Asano et al. reported that the consumption of a sucrose drink containing 10 % (w/w) D-xylose suppressed the rapid elevation of blood glucose and insulin in mice [12]. A sucrose drink containing 10 % (w/w) D-xylose also reduced the glycemic index (GI) by 21.4 % and insulin secretion by 21.3 % in healthy individuals [13].

However, no studies investigated subjects with IFG and the effectivity of a dose of D-xylose less than 10 % (w/w). The current study assesses the acute effect of xylose on postprandial glycemia in subjects with normal glucose levels or prediabetics who consumed sucrose drinks that contained 5 g (Test 1; sucrose:xylose = 10:1), 3.33 g (Test 2; sucrose:xylose = 15:1), or 2.5 g (Test 3; sucrose:xylose = 20:1) of D-xylose with a one-week separation interval.

## Methods

### Study subjects

We posted advertisements in the local newspaper to recruit study subjects, and all clinical tests were conducted in the Clinical Nutrition Research Laboratory at Yonsei University. All study subjects aged 20 to 70 years were classified into two groups, normal and IFG, based on serum glucose levels. Subjects were excluded for the following reasons: 1) history of taking insulin injections or oral hypoglycemic agents, 2) evidence of alcohol abuse or alcoholism, 3) pregnant or breastfeeding, 4) chronic gastrointestinal disorder, 5) seriously abnormal liver or renal function, or 6) an occupation at risk of death when hypoglycemia occurs. A fasting serum glucose level between 100 and 125 mg/dL was defined as IFG. Subjects with fasting glucose levels over 126 mg/dL were newly diagnosed with type 2 diabetes. The Institutional Review Board of Yonsei University approved this study (IRB 163–02), and all participants provided written informed consent. A total of 80 subjects were initially enrolled. Two participants dropped out of the study for personal reasons, and three participants discontinued the study because they were terrified during blood collection. A final total of 25 normal subjects and 50 hyperglycemic subjects were analyzed in this study.

### Study design

We adopted a randomized double-blind, crossover design to determine an effective proportion of D-xylose. All study subjects were randomly assigned using the random assignment table: for the consumption order of four drinks provided by CJ Cheiljedang Co., Ltd. (Guro-Gu, Seoul). The experimental drinks were indistinguishable from each other and included: 1) Control (sucrose 50 g + deionized water 100 g), 2) Test 1 (sucrose:xylose = 10:1, sucrose 50 g + xylose 5 g + deionized water 95 g), 3) Test 2 (sucrose:xylose = 15:1, sucrose 50 g + xylose 3.33 g + deionized water 96.67 g), and 4) Test 3 (sucrose:xylose = 20:1, sucrose 50 g + xylose 2.5 g + deionized water 97.5 g).

Subjects visited the Clinical Nutrition Research Laboratory at Yonsei University for 4–5 weeks and randomly ingested the four test drinks (Control, Test 1, Test 2, and Test 3) with a one-week interval to avoid a carryover effect, which is the persistence and influence of the previous treatment on the following treatment in a crossover design trial [14]. We also asked the subjects to maintain their usual diet and physical activity until the end of the study.

### Anthropometric parameters, blood pressure measurement, and blood collection

The body weight of barefoot subjects was measured on the morning of each visit to obtain the body mass index (BMI, kg/m^2^), which was calculated as body weight (kg) divided by height squared (m^2^). Body fat (%) was determined using a bioelectrical impedance analyzer (InnerScan BC-533, Tanita, Japan), and lean body mass (kg) was calculated using weight (kg) and body fat (%). We also measured waist circumference at the umbilical region and hip circumference at the protruding part of the hip in standing subjects.

Blood pressure (BP) was measured twice in the upper arm after a 5-min rest using a non-invasive blood pressure monitor (EASY X 800, Jawon Medical, Republic of Korea). The average of two BP measurements was recorded. BP was measured a third time when the difference between the two BP measurements was greater than 10 mmHg.

Venous blood specimens were collected in EDTA-treated and plain tubes at five time points: after fasting for 12 h, and 15, 30, 60, and 120 min after the consumption of each test drink. All blood samples were centrifuged to obtain plasma and sera, which were stored in a −70 °C deep freezer until analysis.

### Assessments of dietary intake and physical activity

We investigated subjects’ usual food intake using a 24-h recall method and semi-quantitative food frequency questionnaire (semi-FFQ) during their first visit and instructed participants not to change their usual diet and level of physical activity to avoid any effects on their health and biochemical parameters.

Total calorie intake (TCI, kcal/day), the percentage of calories from carbohydrates, protein, and fat, and the amount of cholesterol in their diet (mg/day) were evaluated using the Computer-Aided Nutritional analysis program (CAN-pro 3.0, Korean Nutrition Society, Korea). Total energy expenditure (TEE, kcal/day) was calculated from the basal metabolic rate (BMR), the 24-h physical activity [15], and the thermic effect of food (TEF). The BMR of each subject was calculated using the Harris–Benedict equation [16].

### Fasting and postprandial glucose, insulin, C-peptide, and FFA concentrations

Venous blood was drawn after a 12-h fast and 15, 30, 60, and 120 min after the consumption of test drinks. The hexokinase method colorimetrically measured NADP to determine fasting and postprandial glucose concentrations in sera using a clinical chemistry analyzer (Hitachi 7600 Autoanalyzer, Hitachi Ltd., Japan). Serum insulin and C-peptide levels were analyzed using commercially available radioimmunoassay (RIA) kits and a gamma counter (Cobra Quantum 5002, Hewlett-Packard, USA). Free fatty acids (FFAs) were measured using an enzymatic method involving acyl-CoA synthetase (ACS) and acyl-CoA oxidase (ACOD).

### Serum lipid profiles and apolipoproteins A-I and B

Fasting triglyceride and total cholesterol concentrations (mg/dL) in sera were measured using enzymatic methods in a clinical chemistry analyzer (Hitachi 7600 Autoanalyzer, Hitachi Ltd., Japan). High-density lipoprotein (HDL) cholesterol (mg/dL) was analyzed in the supernatant using selective inhibition methods after combination with chylomicrons. Low-density lipoprotein (LDL) and very low-density lipoprotein (VLDL) were combined with surfactants. Low-density lipoprotein (LDL) cholesterol (mg/dL) was estimated indirectly using the Friedewald formula. Immunoturbidimetric methods were used to determine serum apolipoproteins A-I and B (mg/dL) using a chemical analyzer (Cobas Integra 400, Roche, Switzerland).

### Safety parameters

The International Federation of Clinical Chemistry (IFCC) recommends the measurement of serum glutamic oxaloacetic transaminase (sGOT or AST) and glutamic pyruvic transaminase (sGPT or ALT) using a kinetic UV method and commercial kits (Roche, Germany) on a chemical analyzer (Hitachi 7600 Autoanalyzer, Hitachi Ltd., Japan). A kinetic UV assay and a colorimetric method, specifically the Jaffe reaction, were used to measure serum creatinine and blood urea nitrogen (BUN), respectively.

A complete blood count (CBC) of white blood cells (10^3^/mm^3^), red blood cells (10^6^/mm^3^), hemoglobin (g/dL), hematocrit (%), and platelets (10^3^/mm^3^) was performed using a hematology analyzer (ABX Micros ES 60, HORIBA ABX Diagnostics, Inc., France).

### Statistical methods

Statistical analyses were performed using SPSS version 18.0 for Windows (Statistical Package for Social Science, SPSS Inc., USA). Each variable was examined for normal distribution, and significantly skewed variables underwent log transformation. The mean values of untransformed variables are presented for descriptive purposes. Baseline characteristics and comparisons between the normal and hyperglycemic groups were evaluated using Student’s *t*-test for continuous variables. Glucose, insulin, and C-peptide areas under the curve (AUCs) were calculated after subtraction of the baseline value from each subsequent measurement using the trapezoidal method [17]. Comparisons between the consumption of the control drink (sucrose 50 g + deionized water 100 g) and each test drink (Test 1: sucrose:xylose = 10:1, Test 2: sucrose:xylose = 15:1, Test 3: sucrose:xylose = 20:1) were evaluated using one-way analysis of variance (ANOVA) and Bonferroni methods at each time point. Only results from participants who completed the intervention program were analyzed. The results are expressed as the means ± standard error of the mean (SEM). We considered results with a *p*-value < 0.05 as statistically significant and results with a *p*-value < 0.1 to exhibit a tendency toward significance.

## Results

### Characteristics of normal and prediabetic subjects

Table 1 shows the general characteristics of the study subjects. Hyperglycemic subjects were older (*p* < 0.001) and had higher concentrations of triglycerides and Apo B than normal subjects. Normal subjects exhibited a higher TEE (kcal/d) than hyperglycemic subjects. There were no significant differences between the two groups in baseline characteristics, such as BMI, body fat, blood pressure, HDL cholesterol, LDL cholesterol, Apo A-I, total energy intake, and the percent energy intake from carbohydrate, protein, and fat.

**Table 1:** Characteristics of study participants

	Normal subjects (n = 25)	Hyperglycemic subjects (n = 50)	*p*-value
n (male/female)	(13/12)	(35/15)	
Age (years)	28.2 ± 1.12	50.1 ± 1.80	<0.0001
Weight (kg)	69.5 ± 2.83	67.7 ± 1.48	0.053
BMI (kg/m^2^)	23.8 ± 0.72	24.4 ± 0.46	0.453
Body fat (%)^a^	25.1 ± 1.24	25.0 ± 1.07	0.742
Lean body mass (kg)^a^	52.1 ± 2.29	50.6 ± 1.18	0.672
Blood pressure (mmHg)			
Systolic BP	119.2 ± 3.45	122.0 ± 2.08	0.470
Diastolic BP^a^	72.1 ± 2.38	74.4 ± 1.42	0.315
Lipids parameters			
TG (mg/dL)^a^	92.3 ± 8.95	133.1 ± 11.58	0.010
T chol (mg/dL)	182.8 ± 6.32	196.4 ± 4.33	0.077
HDL chol (mg/dL)	57.4 ± 1.91	53.5 ± 1.43	0.108
LDL chol (mg/dL)	106.9 ± 5.26	116.5 ± 3.92	0.155
Apo A-I (mg/dL)	156.1 ± 3.52	149.7 ± 3.03	0.204
Apo B (mg/dL)^a^	90.5 ± 5.12	105.5 ± 3.19	0.014
Dietary intake and total energy expenditure			
TEE (kcal/d)	2437 ± 70.8	2281 ± 37.5	0.036
TCI (kcal/d)	2315 ± 70.7	2272 ± 36.5	0.547
Carbohydrate (%)	61.7 ± 0.16	61.6 ± 0.11	0.558
Protein (%)	15.9 ± 0.08	15.9 ± 0.05	0.697
Fat (%)	23.1 ± 0.20	23.0 ± 0.15	0.812
Cholesterol (mg)	188 ± 0.50	186 ± 0.70	0.130

Mean ± SEM

^a^Analyzed after log transformation; tested by Student *t*-test

### Effects of xylose consumption on postprandial glucose metabolism in normal subjects

**Fig. 1:**
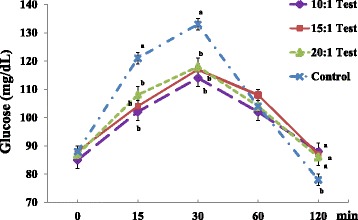
Serum glucose levels in normal subjects. Values are expressed as the means ± SEM. *p*-value tested using one-way analysis of variance (ANOVA) and Bonferroni methods

Normal subjects (n = 25) in all test groups exhibited a significant decrease in serum glucose levels at 15 min (Test 1: 102.3 ± 2.39, Test 2: 104.1 ± 2.76, Test 3: 107.3 ± 2.81 versus Control: 120.8 ± 3.42 mg/dL) and 30 min (Test 1: 113.8 ± 2.40, Test 2: 116.5 ± 2.84, Test 3: 118.2 ± 3.37 versus Control: 132.8 ± 3.74 mg/dL) than the control group (Fig. 1). However, normal subjects exhibited a significant increase in serum glucose levels in all test groups at 120 min compared to the control group. All test groups also exhibited significantly lower serum levels of insulin at 15 min (Test 1: 28.3 ± 5.19, Test 2: 24.8 ± 3.47, Test 3: 31.4 ± 4.05 versus Control: 56.6 ± 7.18 uIU/mL) and 30 min (Test 1: 30.4 ± 3.63, Test 2: 31.0 ± 3.59, Test 3: 35.9 ± 4.66 versus Control: 55.0 ± 5.21 uIU/mL) than the control group (Table 2). The Test 1 group exhibited significantly lower insulin AUC (Test 1: 46.2 ± 5.32 versus Control: 55.0 ± 5.21 uIU/mLxhr). We found significantly lower serum levels of C-peptide at 15 min (Test 1: 3.20 ± 0.31, Test 2: 3.26 ± 0.23 versus Control: 4.67 ± 0.39 ng/dL) and 30 min (Test 1: 4.41 ± 0.31 versus Control: 6.01 ± 0.36 ng/dL) in the test groups compared to the control group.

**Table 2:** Glucose-related markers in normal subjects

		Normal subjects (n = 25)			
		TEST (10:1)	TEST (15:1)	TEST (20:1)	Control
Insulin^a^	0 min	10.6 ± 0.98	9.8 ± 0.85	9.8 ± 0.71	9.3 ± 0.62
uIU/mL	15 min	28.3 ± 5.19^c^	24.8 ± 3.47^c^	31.4 ± 4.05^c^	56.6 ± 7.18^b^
	30 min	30.4 ± 3.63^c^	31.0 ± 3.59^c^	35.9 ± 4.66^c^	55.0 ± 5.21^b^
	60 min	25.8 ± 2.93	29.1 ± 2.35	24.9 ± 2.54	29.0 ± 3.05
	120 min	14.0 ± 1.95^b^	12.0 ± 1.14	12.3 ± 1.04	9.4 ± 0.84^c^
Insulin AUC^a^ (uIU/mLxhr)		46.2 ± 5.32^c^	46.8 ± 3.50	47.4 ± 3.77	62.4 ± 4.91^b^
C-peptide^a^	0 min	1.89 ± 0.17	1.92 ± 0.16	1.82 ± 0.15	1.92 ± 0.14
(ng/mLxhr)	15 min	3.20 ± 0.31^c^	3.26 ± 0.23^c^	3.44 ± 0.26	4.67 ± 0.39^b^
	30 min	4.41 ± 0.31^c^	4.62 ± 0.32	4.82 ± 0.42	6.01 ± 0.36^b^
	60 min	5.15 ± 0.31	5.36 ± 0.31	4.98 ± 0.28	5.70 ± 0.33
	120 min	3.62 ± 0.22^b^	3.49 ± 0.24^b^	3.20 ± 0.21	2.69 ± 0.19^c^
C-peptide AUC^a^ (ng/mLxhr)		8.36 ± 0.48	8.55 ± 0.47	8.23 ± 0.47	9.28 ± 0.50

Mean ± SEM. ^b,c^Tested by ANOVA (Bonferroni)

^a^Analyzed after log transformation

### Comparisons of changes in serum glucose, insulin, and C-peptide levels in normal subjects

**Fig. 2:**
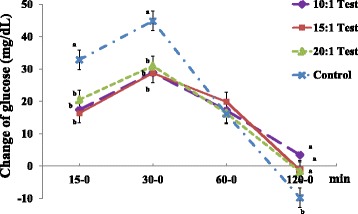
Comparisons of changes in serum glucose levels in normal subjects. Values are expressed as the means ± SEM. *p*-value tested using one-way analysis of variance (ANOVA) and Bonferroni methods

All test groups exhibited significantly lower changes in serum glucose levels at 15–0 (Test 1: 17.4 ± 2.28, Test 2: 16.4 ± 2.23, Test 3: 20.5 ± 2.75 versus Control: 32.9 ± 3.24 mg/dL) and 30–0 (Test 1: 28.9 ± 2.07, Test 2: 28.8 ± 2.58, Test 3: 31.0 ± 3.03 versus Control: 44.9 ± 3.02 mg/dL) compared to the control group (Fig. 2). We also found significantly lower changes in serum insulin and C-peptide levels at 15–0 and 30–0 compared to the control group (data not shown).

### Effects of xylose consumption on postprandial glucose metabolism in hyperglycemic subjects

**Fig. 3:**
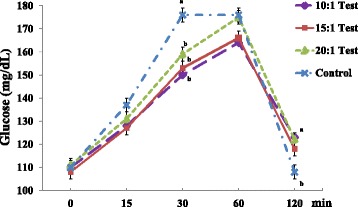
Serum glucose levels in hyperglycemic subjects. Values are expressed as the means ± SEM. *p*-value tested using one-way analysis of variance (ANOVA) and Bonferroni methods

Hyperglycemic subjects (n = 50) in the test groups exhibited a significant decrease in serum glucose levels at 30 min (Test 1: 149.6 ± 3.44, Test 2: 152.5 ± 3.56, Test 3: 158.8 ± 3.99 versus Control: 176.4 ± 5.18 mg/dL) compared to the control group (Fig. 3). However, the Test 1 group exhibited a significant increase in serum glucose levels at 120 min compared to the control group. The glucose-related markers did not significantly differ between groups (Table 3).

**Table 3:** Glucose-related markers in hyperglycemic subjects

		Hyperglycemic subjects (n = 50)			
		TEST (10:1)	TEST (15:1)	TEST (20:1)	Control
insulin^a^	0 min	10.0 ± 0.77	10.4 ± 1.13	10.9 ± 1.39	9.5 ± 0.57
uIU/mL	15 min	15.2 ± 1.41	16.4 ± 1.57	17.5 ± 1.95	21.6 ± 1.75
	30 min	24.5 ± 2.31	24.6 ± 2.72	25.2 ± 2.48	33.3 ± 3.19
	60 min	30.8 ± 2.80	29.5 ± 3.41	33.7 ± 3.74	35.7 ± 4.46
	120 min	19.7 ± 3.59	20.2 ± 3.48	21.7 ± 4.36	16.1 ± 2.74
insulin AUC^a^ (uIU/mLxhr)		47.2 ± 4.69	46.9 ± 5.34	51.8 ± 6.24	53.9 ± 5.58
C-peptide^a^	0 min	2.43 ± 0.13	2.40 ± 0.14	2.53 ± 0.16	2.42 ± 0.12
(ng/mLxhr	15 min	3.12 ± 0.14	3.21 ± 0.14	3.39 ± 0.17	3.72 ± 0.19
	30 min	4.30 ± 0.22	4.31 ± 0.23	4.52 ± 0.20	5.15 ± 0.27
	60 min	5.87 ± 0.27	5.89 ± 0.30	6.37 ± 0.29	6.51 ± 0.39
	120 min	5.32 ± 0.31	5.48 ± 0.31	5.66 ± 0.32	4.78 ± 0.31
C-peptide AUC^a^ (ng/mLxhr)		9.76 ± 0.44	9.87 ± 0.46	10.46 ± 0.46	10.44 ± 0.53

Mean ± SEM. Tested by ANOVA (Bonferroni)

^a^Analyzed after log transformation

### Comparisons of changes in serum glucose, insulin, and C-peptide levels in hyperglycemic subjects

**Fig. 4:**
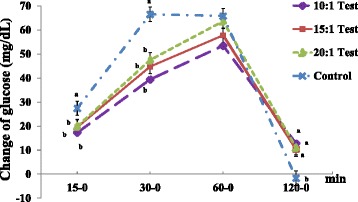
Comparisons of changes in serum glucose levels in hyperglycemic subjects. Values are expressed as the means ± SEM. *p*-value tested using one-way analysis of variance (ANOVA) and Bonferroni methods

The test 1 group exhibited a significantly lower change in serum glucose levels at 15–0 (Test 1: 17.3 ± 2.11 versus Control: 27.4 ± 2.79 mg/dL), and all test groups exhibited significantly lower changes in serum glucose levels at 30–0 (Test 1: 39.4 ± 2.47, Test 2: 44.8 ± 2.48, Test 3: 47.5 ± 2.55 versus Control: 66.5 ± 4.05 mg/dL) than the control group (Fig. 4). We also found significantly lower changes in serum insulin and C-peptide levels at 15–0 and 30–0 compared to the control group (data not shown).

### Safety parameters of normal and prediabetic subjects

Table 4 shows the safety parameters of study subjects. Hyperglycemic subjects exhibited higher concentrations of GOT and GPT than normal subjects. However, these concentrations were within normal ranges.

**Table 4:** Safety parameters

	Normal subjects (n = 25)	Hyperglycemic subjects (n = 50)	*p*-value
WBC (х10^3^/uL)	5.59 ± 0.25	5.82 ± 0.18	0.468
RBC (х10^3^/uL)	4.28 ± 0.08	4.33 ± 0.05	0.558
HGB (g/dL)	13.1 ± 0.37	13.4 ± 0.14	0.543
HCT (%)	40.7 ± 1.08	41.8 ± 0.42	0.382
PLT (х10^3^/mm^3^)	256 ± 13.3	244 ± 5.69	0.398
GOT (U/L)	19.7 ± 0.83	25.3 ± 1.09	<0.001
GPT (U/L)	14.8 ± 1.29	25.3 ± 2.44	<0.001
BUN (mg/dL)	12.4 ± 0.52	14.2 ± 0.55	0.028
Creatinine (mg/dL)	0.76 ± 0.03	0.75 ± 0.02	0.957

Mean ± SEM. tested by Student *t*-test

## Discussion

The present study determined the effects of xylose supplementation on postprandial glycemic responses in normal and hyperglycemic subjects. We investigated whether D-xylose less than 5 g per 50 g sucrose exerted beneficial effects on postprandial glycemic control. No adverse biochemical effects of D-xylose were observed in any subject during the entire study. We found that xylose supplementation may exhibit beneficial effects on postprandial glucose metabolism. Notable effects on postprandial glucose, insulin, and C-peptide levels were observed 15 and 30 min after the consumption of test drinks.

Xylose is a white crystalline sugar that exhibits approximately 60 % of the sweetness of sucrose. Various plants, including coconut and straw, are rich sources of D-xylose [13]. Administration of a sucrose solution containing 10 % D-xylose (w/w) inhibited the rapid elevation of blood glucose in rats [12]. Bae et al. reported that the consumption of sucrose drinks containing 10 % D-xylose (w/w) reduced GI 21.4 % and insulin secretion 21.3 % in healthy individuals [13]. Bae et al. reported on the inhibitory effects of two different concentrations of xylose (5 and 7.5 g) on postprandial serum glucose and insulin concentrations in healthy subjects. However, our study examined the effect of three different concentration of xylose (2.5, 3.33, and 5 g) on postprandial serum glucose and insulin concentrations in subjects with normal glucose levels and hyperglycemia. We focused on the effect of low concentrations of xylose.

Some studies examined whether sucrose drinks with 5 % (w/w) D-xylose exerted beneficial effects on enhancing the glycemic response [18, 19], but the number of subjects in these studies was too small (n = 10 and n = 13) to define the effect of D-xylose.

The homeostasis of glucose and other glucose-related metabolites, such as insulin and C-peptide, were the targets for the prevention of metabolic syndrome, such as type 2 diabetes and cardiovascular disease [13]. Glucose absorption in the intestines should be adequately regulated to maintain glycemic homeostasis. Therefore, it is important that sucrase not rapidly hydrolyze sucrose into glucose and fructose. The effect of D-xylose occurs via the inhibition of sucrase activity in a non-competitive manner [12]. Specifically, D-xylose inhibits sucrase activity by combining with the intermediate of sucrase and glucose, immediately after the dissociation of fructose [18].

However, the ability of D-xylose to control the postprandial glycemic response may be difficult to sustain because a large amount of xylose is excreted rapidly in urine [20]. Significant results in the prevention of the rapid increase in blood glucose, insulin, and C-peptide were observed at 15 min and 30 min, but this effect was not sustained at the later time points of 60 min and 120 min. Blood glucose levels were even higher 120 min after the consumption of xylose-containing drinks compared to a control drink, which may have occurred because secretion of the glucose-lowering hormone insulin was less stimulated by lower levels of serum glucose at 15 min and 30 min. However, xylose still effectively controlled postprandial glycemic because the AUCs of glucose, insulin, and C-peptide were significantly smaller after the consumption of all test drinks containing xylose.

One limitation of this study is that it evaluated the inhibitory effect of xylose on postprandial hyperglycemia after the intake of sucrose, but not complex carbohydrates. Our study period was also very short, and all of the test products were consumed once weekly. Therefore, further studies are required to identify how long-term xylose consumption affects glucose control and the effect of postprandial glucose on complex carbohydrates containing xylose (for example, muffins or sandwiches).

The present study suggests that xylose supplementation (5, 3.33, and 2.5 g) in drinks exhibits a beneficial effect on postprandial glycemic response and glucose-related biomarkers, including insulin and C-peptide levels, in subjects with normal glucose levels and impaired fasting glucose.

## Conclusions

We investigated whether D-xylose less than 5 g per 50 g sucrose exerted beneficial effects on postprandial glycemic control. We found that xylose supplementation may exert beneficial effects on postprandial glucose metabolism. Notable effects on postprandial glucose, insulin, and C-peptide levels were observed 15 and 30 min after the consumption of test drinks. Xylose supplementation may exert a beneficial effect on postprandial glycemic responses in subjects with normal glucose levels and prediabetes.

## Abbreviations

AUC: area under the curve
BMI: body mass index
BP: blood pressure
FFA: free fatty acids
HbA1c: hemoglobin A1c
HDL: high-density lipoprotein
IFG: impaired fasting glucose
IGT: impaired glucose tolerance
LBM: lean body mass
LDL: low-density lipoprotein
OGTTs: oral glucose tolerance tests
T2DM: type 2 diabetes mellitus
TG: triglycerides
WHR: waist-to-hip ratio
